# Longitudinal conductivity of LaF_3_/SrF_2_ multilayer heterostructures

**DOI:** 10.1080/14686996.2016.1246940

**Published:** 2016-11-25

**Authors:** Tikhon Vergentev, Alexander Banshchikov, Alexey Filimonov, Ekaterina Koroleva, Nikolay Sokolov, Marc Christopher Wurz

**Affiliations:** ^a^ Institute of Physics, Nanotechnology and Telecommunications, Peter the Great St. Petersburg Polytechnic University, Saint-Petersburg, Russia; ^b^ Divisions of Solid State Physics and Physics of Dielectric and Semiconductors, Ioffe Institute, Saint-Petersburg, Russia; ^c^ Institute for Microproduction Technology, Leibniz University of Hanover, Garbsen, Germany

**Keywords:** Impedance spectroscopy, ionic conductivity, lanthanum fluoride, strontium fluoride, molecular beam epitaxy, heterostructures, longitudinal conductivity, interfacial spacing, 40 Optical, magnetic and electronic device materials, 103 Composites, 105 Low-Dimension (1D/2D) materials, 212 Surface and interfaces, 306 Thin film /Coatings

## Abstract

LaF_3_/SrF_2_ multilayer heterostructures with thicknesses of individual layers in the range 5–100 nm have been grown on MgO(100) substrates using molecular beam epitaxy. The longitudinal conductivity of the films has been measured using impedance spectroscopy in the frequency range 10^−1^–10^6^ Hz and a temperature range 300–570 K. The ionic DC conductivities have been determined from Nyquist impedance diagrams and activation energies from the Arrhenius–Frenkel equation. An increase of the DC conductivity has been observed to accompany decreased layer thickness for various thicknesses as small as 25 nm. The greatest conductivity has been shown for a multilayer heterostructure having thicknesses of 25 nm per layer. The structure has a conductivity two orders of magnitude greater than pure LaF_3_ bulk material. The increasing conductivity can be understood as a redistribution of charge carriers through the interface due to differing chemical potentials of the materials, by strong lattice-constant mismatch, and/or by formation of a solid La_1-x_Sr_x_F_3-x_ solution at the interface during the growth process.

## Graphical abstract

**Figure:**
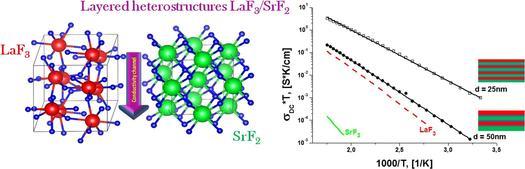

## 1. Introduction

Combinations of *M*F_2_ and *R*F_3_ fluorides (*M*- alkaline-earth and *R*- rare-earth elements) are perspective materials that demonstrate high ionic conductivity.[1] Growing of superionic materials by molecular beam epitaxy (MBE) allows creation of composite materials with defined thicknesses and physical properties; this is useful not only for decreasing power consumption of devices,[2] but also for cardinally varying physical properties of materials. Based on this growth technique, fluoride sensors,[3] oxygen sensors,[4] batteries,[5] and transistors [6] have been proposed. In addition, these growth studies offer a good possibility to study the nature of fast ionic transport, the influence of size effects on conductivity, and surface interactions in nanoparticles or films.

Maier et al. [7] have studied the influence of interface interactions between BaF_2_ and CaF_2_ films on conductivity. They have demonstrated that the longitudinal conductivity increases by two orders of magnitude in comparison with the longitudinal conductivity of pure components. BaF_2_/CaF_2_ is considered as a model system to study *M*F_2_/*M*′F_2_ multilayers based on their structure features and conductivities. Several approaches for calculating the transport properties of these systems can be found in the literature. One of these approaches involves the consideration of heterostructures as a combination of individual layers with fluorite ion enrichment near to an interface. Enrichment is caused by a relative motion of ions ${\text{F}}^{-}$ and vacancies ${\text{V}}_{{\text{F}}^{-}}$ through the boundary of materials, which influences the concentration profiles.[8] Such a model aids in understanding the mechanism of increasing longitudinal conductivity along the interfaces.[9–11] A formation of solid solution nearby the interface is also possible.[12] The conductivity of the interface will be higher than the conductivity of the initial materials. As we have recently shown,[13] the ionic conductivity of LaF_3_ films on CaF_2_(111) and MgO(100) substrates is completely different, which is due to the additional interaction between the film and the ionic fluoride substrate. The study of composite materials with a combination of different phases, structures, and conductivities will likely be valuable in elucidating the modification of ionic transport properties.

Our previous papers demonstrate some aspects of ionic transport, dependent on the pore size [14] in doped materials, stoichiometry of films of solid solutions,[15] and magnitude of ionic conductivity in films grown on different substrates.[13] Herein, we investigate longitudinal conductivity of LaF_3_/SrF_2_ multilayer heterostructures with different individual layer thicknesses and a constant total thickness (200 nm) grown on MgO(100) substrates. Lanthanum fluoride with tysonite structure and solid solutions LaF_3_-SrF_2_ (La_1-x_Sr_x_F_3-x_) are extensively studied because if their high ionic conductivities. Heterovalent replacements of Sr^+2^ and La^+3^ ions in tysonite LaF_3_ cells promote the exchange of charge carriers and increase their mobility. Other physical mechanisms may be responsible for the observed increase in conductivity at the interfaces of the heterostructures. We expect that the production of heterostructures based on fluoride materials with different LaF_3_/SrF_2_ crystal structure could be interesting not only for applications but also as a subject for fundamental studies. Such structures may demonstrate a greater increase of conductivity as a function of layer thickness than BaF_2_/CaF_2_ heterostructures with consideration of the layer thickness.

## 2. Experimental details

Films were grown on epi-ready MgO(100) substrates by the MTI company (Richmond, USA) using the MBE method in an ultra-high vacuum chamber equipped with reflection high-energy electron diffraction (RHEED). Surface roughness was quoted in the substrate manufacturer’s technical datasheets as R_z_ < 10 Å. MgO(100) substrates had a size of 3 × 10 × 0.5 mm^3^. Prior to the coating, the substrates were fixed on the electrical heater and were annealed at a temperature of 1200 °C. The method of surface preparation was identical for all measured substrates. Tests showed no difference on electrical and structural properties pre- and post-annealed substrates MgO(100). LaF_3_/SrF_2_ heterostructures and films of solid solutions were grown in a base vacuum of 10^−8^ Pa. The temperature of the substrates was maintained at 750 °C during the growth process. The thicknesses of the films were measured with a quartz crystal microbalance with 5% precision. The average growth rate was about 2 nm min^–1^. The gold electrodes for conductivity measurements were deposited at the end of the samples through a nickel mask. The intermediate and final crystal structures were monitored by RHEED and X-ray diffraction (XRD). Samples were characterized by atomic-force microscopy (AFM) and scanning electron microscopy (SEM) studies.

Electrical properties were studied using a dielectric spectrometer Novocontrol BDS`80 (Novocontrol Technologies GmbH & Co. KG, Germany) in the temperature range 300–570 K and a frequency range of 10^−1^ –10^6^ Hz. Conductivity was measured parallel to the interfaces of multilayer structures. The distance between the electrodes was 2 mm and the electric field value was E = 5 V cm^–1^. Temperature-resolved measurements were carried out at a heating rate of 1 K min^–1^.

Multilayer heterostructures with a total thickness D = 200 nm were studied. Thickness of each layer is *d* = D/k, where k = 2, 4, 6, 8, 10, 14, 20, 30, and 40, referred afterwards as interfacial spacing.[11] Each layer inside a LaF_3_/SrF_2_ heterostructure has the same thickness. The first layer on the MgO(100) substrate is LaF_3_ for all samples. Films of La_0.95_Sr_0.05_F_2.95_ and La_0.5_Sr_0.5_F_2.5_ solid solutions as well as pure LaF_3_ and SrF_2_ with 200 nm thickness were grown on MgO(100) substrates and were studied under the same conditions as the multilayer heterostructures.

## 3. Results and discussion

**Figure 1.**
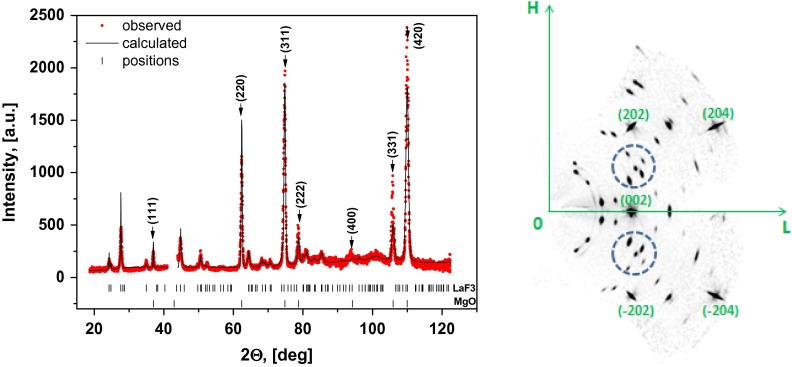
XRD pattern (left panel) obtained by angular integration of the map recorded in the (HKL) reciprocal space. Features marked by vertical arrows (↓) originate from the MgO substrate, while the other peaks belong to the LaF_3_ film. The region from 42° to 44° was excluded to avoid the strong (200) MgO peak. The right panel shows a reciprocal space layer corresponding to (H0L) plane. Indexed nodes are characterized by MgO cubic structure and the intermediate nodes are characterized by LaF_3_ film.

**Figure 2.**
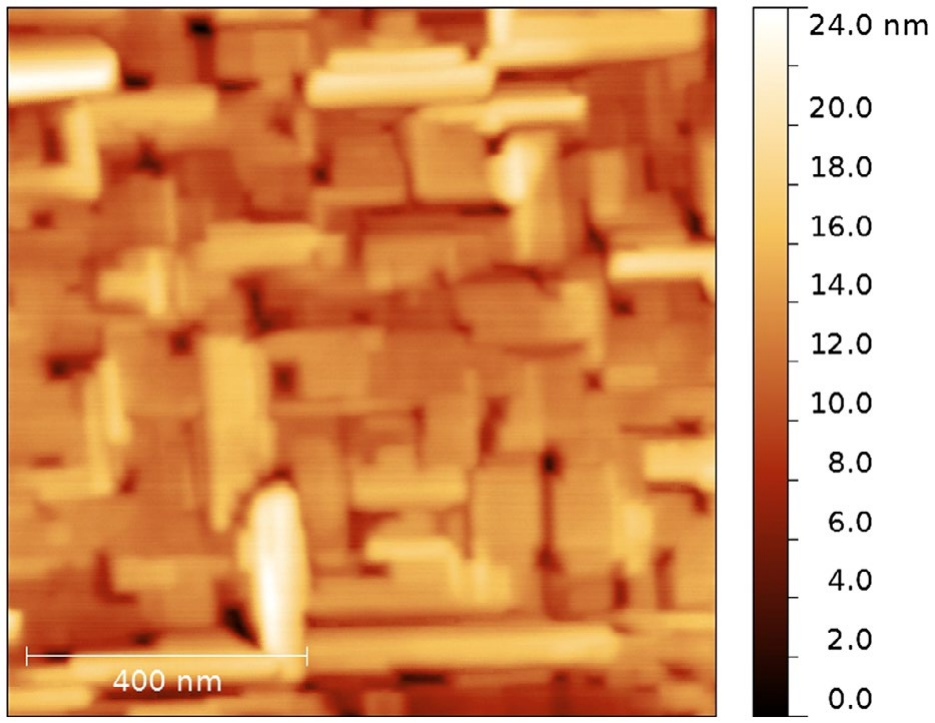
AFM image of a 200-nm thick LaF_3_ film.

XRD analysis was performed using a single-crystal X-ray diffractometer SuperNova equipped with a CCD detector (Rigaku Oxford Diffraction, Great Britain), λ_Cu_ = 1.54184 Å. The sample was rotated in its plane. XRD pattern obtained by angular integration of the maps recorded in the (HKL) reciprocal space presented in Figure 1, left panel. The resulting XRD pattern was processed with the standard profile analysis program FullProf.[16] Features marked by vertical arrows (↓) originate from the MgO substrate, while the other peaks belong to the LaF_3_ film. The region from 42° to 44° was excluded to avoid the strong (200) MgO peak. The LaF_3_ film on the MgO (100) has hexagonal structure with the cell parameters *a* = *b* = 7.1810 Å, *c* = 7.3296 Å, *α* = *β* = 90°, *γ* = 120°. The right panel in Figure 1 shows a reciprocal space layer corresponding to (H0L) plane. Its strongest peaks originate from the substrate, while numerous weaker features belong to the film. Splitting of Bragg peaks from the film (see e.g. reflections inside the marked circles) correspond to the twin structure of the film. The mosaic structure observed in atomic force microscopy (AFM) images (Figure 2) also indicates to possible twinning. Here we observe mosaic block structure with the size about hundreds of nm and the preferred relative orientation about 90° (e.g. when *a* and *c* axis are reversed). Small disorientation of the blocks leads to a smearing of reflections from the film (see reflections on right panel of Figure 1). Occurrence of merohedral twins in LaF_3_ bulk crystals was previously reported in [17]. The lattice matching of a substrate and a film is $100\%\cdot \left(\frac{2\cdot a_{MgO}}{c_{{\text{LaF}}_{3}}}-1\right)≃13\%$, where *a*_*MgO*_ is the lattice parameter of MgO cubic crystal structure (~4.2 Å) and $c_{{\text{LaF}}_{3}}$ is the *c*-axis of hexagonal LaF_3_ structure.

All the studied heterostructures had LaF_3_ as the bottom layer and their structure is described above. Their next layer is SrF_2_ with a cubic structure, which was characterized by RHEED. The difference of lattice parameters is $100\%\cdot \left(\frac{\sqrt{2}\cdot a_{{\text{SrF}}_{2}}}{c_{{\text{LaF}}_{3}}}-1\right)≃10\%$, where $a_{{\text{SrF}}_{2}}$is the lattice parameter of SrF_2_ cubic crystal structure (~5.8 Å). Structural properties were similar for the LaF_3_ layers grown either on SrF_2_ or on MgO(100). RHEED analysis showed that each layer had crystalline structure and that all LaF_3_ layers and all SrF_2_ layers had the same structures.

**Figure 3.**
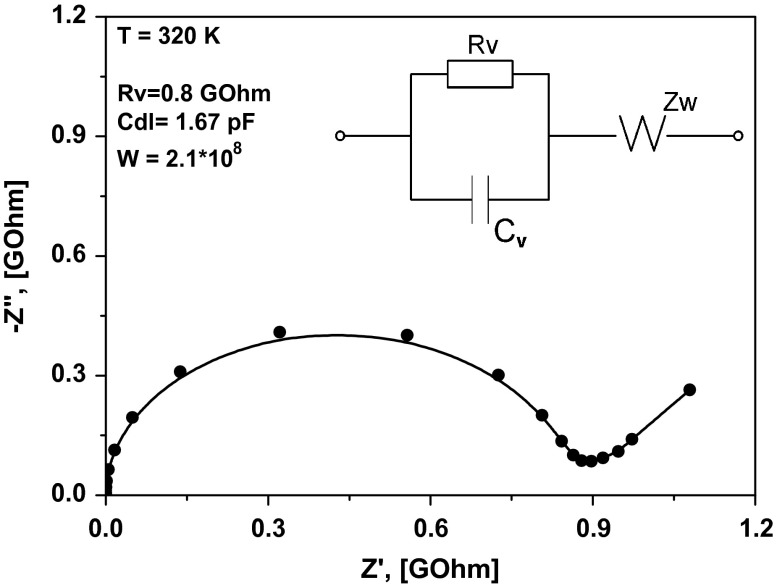
Nyquist plot for the heterostructure with *d* = 20 nm measured at 320 K (solid circles). The inset shows the equivalent circuit that was used in the fit (solid line).

Direct current (DC) conductivities can be obtained from the alternating current (AC) measurements using a spectrum of impedance at each temperature point. The spectrum of impedance is presented as a $Z^{\prime \prime }\left(\omega \right)={\left.f\left(Z^{\prime }\left(\omega \right)\right)\right|}_{T=\text{const}}$ function called a Nyquist plot or hodograph of impedance, where $Z^{\prime \prime }\left(\omega \right)$ is the imaginary part of complex impedance Z* and $Z^{\prime }\left(\omega \right)$ is the real part of the impedance. A typical form of the function for solid ionic electrolytes with one relaxation process consists of a semicircle and a sloped line.[18] Figure 3 illustrates the spectrum of impedance for LaF_3_/SrF_2_ heterostructures with thicknesses of 20 nm of each layer. The inset in the figure demonstrates a typical equivalent circuit, which describes the electrical properties of the measured samples. In the conventional case, the semicircle is described by an RC circuit, where *R*_*v*_ is the bulk resistance of the sample and *C*_*v*_ is its capacitance. The straight line, appearing at low measured frequencies, corresponds to the diffusion of charge carriers to the electrode boundary; this behaviour can be often approximated by a Warburg function *Z*_*w*_ = (1 – *j*)*Wω* ^−0*.*5^.[19] The point of the intersection of the semicircle with the horizontal axis is determined by the DC-resistance of the sample (*R*_*v*_). Hence, DC conductivities are calculated as $\sigma_{DC}=\frac{1}{R_{V}}\frac{d}{S}$, where *d* is the distance between the electrodes and *S* is the square of section under the electrode covering 200 nm of heterostructure. In our case, *S* is a multiplication of the film thickness and the sample length. Herein, the square of the substrate under the electrode is not taken into account because of the resistance of MgO bulk material, which is much greater than the resistance of ionic films.

**Figure 4.**
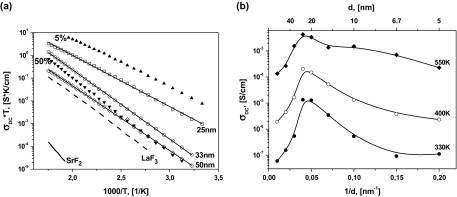
(a) Temperature dependencies of longitudinal conductivity of SrF_2_ (solid lines) and LaF_3_ films (dashed lines) heterostructures with *d* = 25, 33, and 50 nm (open squares, circles, and rhombuses, respectively) and films of solid solutions La_1-x_Sr_x_F_3-x_ with 5% and 50% SrF_2_ content (full triangles). The lines are fits to the Arrhenius–Frenkel equation. (b) The dependence of the longitudinal conductivity on 1/d at 330, 400, and 550 K.

Temperature dependences of DC conductivities are found from the hodograph of impedance, and presented as a *σT* vs. 1000/*T* plot in Figure 4(a). The conductivity of LaF_3_/SrF_2_ multilayer heterostructures is increased by two orders of magnitude in comparison with pure LaF_3_ bulk material. For comparison, similar dependences for the DC conductivity of pure materials (LaF_3_, SrF_2_) are shown in Figure 4(a). This may be caused by an increase in conductivity along the boundaries of layers and an increase of the interface contribution to the total conductivity.

The observed increase of the longitudinal conductivity of heterostructures extends to an interfacial spacing of 25 nm. This can be visualized as a function of the interfacial spacing, *σ*(*d*) or *σ*(1/*d*) (Figure 4(b)). This abrupt increase of the conductivity is probably caused by highly conductive thin layers between LaF_3_ and SrF_2_. Similar behaviour of conductivity was observed by Maier et al. [7] in CaF_2_/BaF_2_ heterostructures and was attributed to the presence of space charge regions at the interfaces. In the case of an ultrathin film of oxide ion conductor the conductivity of epitaxial YSZ (ZrO_2_ + 10%Y_2_O_3_) thin film increased about 150 times when thickness decreased from 60 to 15 nm,[20] attributed to the residual stress caused by misfit in crystal lattice of film and substrate. The main reason for increased conductivity in defective perovskite oxides could be assigned to an extended lattice which is introduced by epitaxial growth of the film.[21, 22]

Further decrease in the interfacial spacing to less than 20 nm decreases the longitudinal conductivity, probably because of the overlapping interface layers and an influence of the film roughness. Note that the roughness of a 200 nm heterostructure is about 20 nm, as estimated using AFM. The decrease of conductivity is accompanied by a change of the impedance spectrum, where the semicircle moves below the abscissa Re(Z). In this case, the equivalent circuit is described with a constant-phase element (CPE) instead of a capacitor.[19] It can be approximated with Cole–Cole function $\sigma \left(\omega \right)=\sigma_{\infty }+\frac{\sigma_{0}-\sigma_{\infty }}{1+{\left(j\omega \tau \right)}^{m}}$, where *m* deviates from a standard Debye spectrum.[13, 14, 23] For the heterostructures with interfacial spacing down to 20 nm, the factor *m* is decreased from 1 to 0.85. Such changes are probably caused by strong mechanical stresses on the crystal lattice, which influences the lattice polarization and the spectrum of the impedance. We do not expect that the interface roughness can significantly affect the shape of the relaxation spectrum.

**Figure 5.**
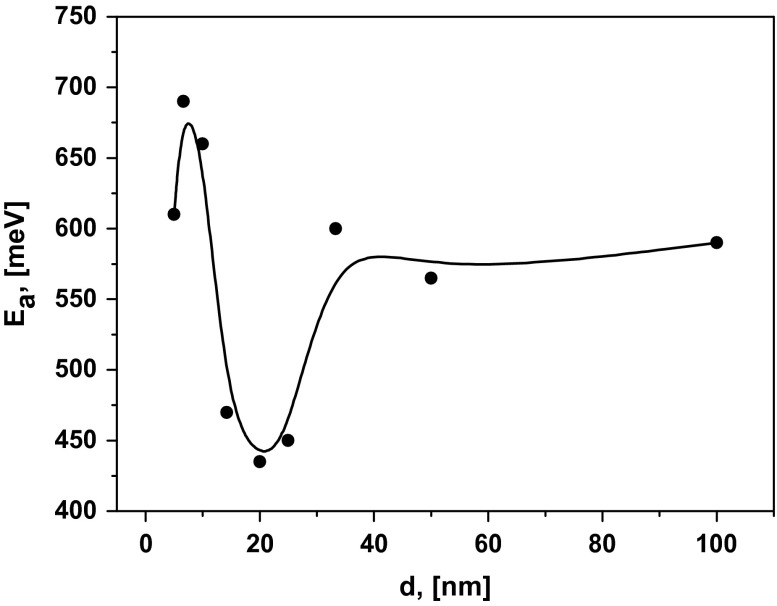
The dependence of activation energy (*E*_*a*_) on interfacial spacing (*d*).

The lines in Figure 4(a) represent a fit to the Arrhenius–Frenkel function $\sigma_{DC}T=\sigma_{0}\cdot \text{exp}\;\left(-\frac{E_{a}}{kT}\right)$, where *E*_*a*_ is the activation energy and *σ*_0_ is the pre-exponential factor. Each line is well characterized by a single slope or activation energy in the entire investigated temperature range. This we can conclude there is no change of the ion transport mechanism [24] in this temperature range. The dependence of *E*_*a*_ on the layer thickness is presented on Figure 5. The curve has a minimum near 450 meV for the multilayer heterostructures with *d* = 15–25 nm. The activation energy decreased from 600 to 450 meV when the thickness *d* was reduced from 100 to 20 nm. Similar behaviour of *E*_*a*_ was reported previously in oxygen-ion conductor films with large lattice mismatch at the interface. In epitaxial YSZ thin films on MgO (mismatch ~18%) the activation energy decreased from 1.09 to 0.62 eV when the YSZ thickness was reduced from 60 to 15 nm.[20, 25] It should be noted that the activation energies of the epitaxial perovskite-type oxide proton conductor SrZr_0.95_Y_0.05_O_3_/SrTiO_3_ on MgO(001) show relatively higher value than the corresponding single crystal in substrates.[26, 27] Also, a substantial change in the activation energy can be observed when creating solid solutions La_1-x_Sr_x_F_3-x_ with heterovalent replacement.[28–30] It is known that La_1-x_Sr_x_F_3-x_ solid solutions with Sr^+2^–La^+3^ replacements in the LaF_3_ cell have greater conductivity than pure LaF_3_ and lower activation energy. As has been shown previously, films of La_1-x_Sr_x_F_3-x_ on glass ceramic substrates have a maximum conductivity at *x* = 0.05.[15] It may be illustrative to compare the conductivity and activation energy of solid solutions and multilayer heterostructures. The conductivities of La_0.95_Sr_0.05_F_2.95_ (*E*_a _= 420 meV) and La_0.5_Sr_0.5_F_2.5_ (*E*_a_ = 700 meV) solid solutions and 25-nm heterostructure (*E*_a _= 450 meV) are presented in Figure 4(a). The 25-nm heterostructure, which has the highest conductivity for heterostructures, exhibits a conductivity that is two orders of magnitude greater at room temperature and one order of magnitude greater at 570 K compared with films of La_0.5_Sr_0.5_F_2.5_ solid solution. However, its conductivity is one order of magnitude less than that of La_0.95_Sr_0.05_F_2.95_ solid solution. The activation energy of 25-nm heterostructure and 5% solid solution are very close. SEM imaging of the heterostructure cross-section allows detailed study of the contrast of each material. However, SEM resolution is not sufficient for studying the interface features of heterostructures. Substantial mixing of the phases is not observed but the data are not inconsistent with a solid solution formation on the interface. We have estimated the thickness of the interface layer of 5% solid solution (which has the greatest conductivity for solid solutions), which would allow us to obtain the conductivity as in the heterostructures. In this case, the thickness should be about 3 nm for each layer, which seems unlikely at our temperatures and durations of growth. Unfortunately, we were not able to determine experimentally the chemical composition variation as a function of the sample depth with the required precision, as the roughness of our film is about 20 nm.

The dependence of conductivity on the single-layer thickness of our structures was calculated using the approach of Maier et al. [7–11]. The differences in chemical potential can affect the redistribution of charge carriers (F^–^) through the interface such that the conductivities of the interface and of the bulk are different. The layer-thickness effects on the ratio of bulk and surface conductivities and the part corresponding to the bulk conductivity vanishes at small thicknesses. If the assumption that all charge carriers are mobile in equilibrium (the Gouy–Chapman case) and the condition of electro neutrality of the bulk is true, the solution of the Poisson–Boltzmann relationship [31, 32] has been obtained. The temperature dependence of mobile ion concentrations can be estimated as $n=\frac{6\sigma_{DC}kT}{q^{2}l^{2}\upsilon }$, where *q* is the electron charge, *l* is the jump distance, and *υ* is the hopping frequency of mobile ion jumps, which can be found from the frequency dependence of the conductivity *σ*(*ω)* as described in detail in [28]. The value of $l_{{\text{LaF}}_{3}}$ is the minimum distance between the closest positions in the LaF_3_ structure ∽2.54 Ǻ. As the conductivity of LaF_3_ can be approximated by:

(1) $$\sigma T=4.115\times 10^{4}e^{-\frac{7258}{T}}\;\left[\frac{S*K}{\text{cm}}\right]$$

and hopping frequency of mobile ion jumps as:

(2) $$\nu_{{\text{LaF}}_{3}}=8.251\times 10^{13}e^{-\frac{4986}{T}}\;\left[s^{-1}\right],$$

the concentration of charge carriers in the bulk of LaF_3_ is:

(3) $$n_{{\text{LaF}}_{3}}=2.5\times 10^{21}e^{-\frac{2272}{T}}\;\left[{\text{cm}}^{-3}\right],$$

with $n=n_{F_{i}^{{}^{\prime }}}=n_{V\cdot_{F}}$ referring to bulk material.

The estimated value of the Debye length at 300 K is $\lambda_{{\text{LaF}}_{3}}$ = 2.8 nm. Thus, all studied structures (up to 10 nm/layer) are characterized by large spacings d>4λ. Using the solution of the Poisson equation for large spacings we have:

(4) $$\sigma^{\left|\right|}\left(d\right)=\sigma_{\infty }+\frac{2}{d}\mu \sqrt{2\varepsilon \varepsilon_{0}kTn_{0}},$$

where

(5) $$n_{0}=n_{\infty }e^{-\frac{zq\cdot \Delta \varphi }{kT}},$$

*z* is the charge number, which equals –1 for $F_{i}^{{}^{\prime }}$ and +1 for $V_{F}^{\cdot }$, and Δ*φ* = *φ*_∞_ − *φ*_0_ is a fitting parameter, which is a difference of space charge potentials.[10] One may also use an expression only including the vacancy mechanism of the LaF_3_ component:

(6) $$\;\sigma^{\left|\right|}\left(d\right)≅\sigma_{\infty ,{\text{LaF}}_{3}}+\sigma_{V_{F}^{\cdot },\;\;{\text{LaF}}_{3}}^{\left|\right|}$$

based on two factors: (1) that the conductivity in SrF_2_ layer is negligible; and (2) that $V_{{\text{F}}^{-}}$ are the main charge carriers in the LaF_3_ layer from the analogy to solid solutions, in which the 5% SrF_2_ substitution shows the significantly greater conductivity than in LaF_3_.

**Figure 6.**
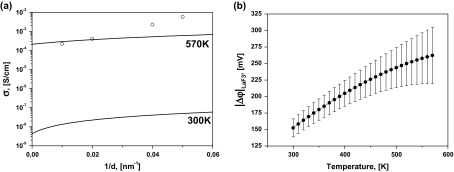
(a) The dependence of the longitudinal conductivity vs. 1/*d* at 330 and 550 K. Markers represent the conductivity of heterostructures with *d* = 25, 33, 50, and 100 nm at 300 K (triangles) and 570 K (circles) and lines were generated by using Equations (4)–(6). (b) Temperature dependence of the fitting parameter Δ*φ* with the standard errors.

**Figure 7.**
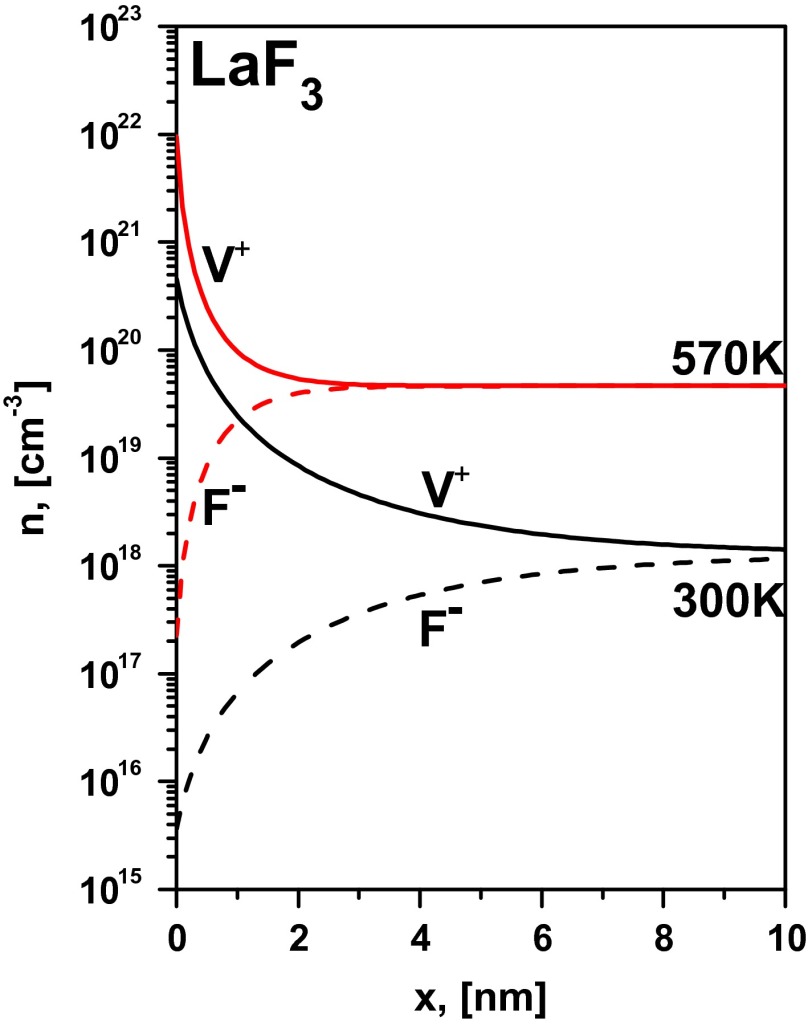
Concentration profiles of charge carriers in LaF_3_ layer in the Gouy–Chapman model for large spacing.

Figure 6(a) presents the longitudinal conductivity as a function of inverse interfacial spacing, where the plot markers are the experimental data and the lines are the fitting using Equations (4)–(6). The temperature dependence of the fitting parameter ${\left|\Delta \varphi \right|}_{{\text{LaF}}_{3}}$ is presented in Figure 6(b). The difference of space charge potentials (${\left|\Delta \varphi \right|}_{{\text{LaF}}_{3}}$) increases from 150 mV (at 300 K) to 250 mV (at 570 K). The corresponding calculated concentration profiles of mobiles carriers are presented in Figure 7. It should be noted that the conductivity mechanism in LaF_3_ is determined by vacancies and the concentration of fluorine vacancies at the boundary increases by almost three orders of magnitude.

This model describes quite well the conductivity behaviour for thick layers (100 and 50 nm), but for 33- and 25-nm layers we have observed a stronger increase of conductivity. As in the case of doped oxides,[33] the enhancement of heterostructure conductivity cannot be explained using only the formation of a space-charge region at the interface; structural effects due to lattice mismatch need to be taken into account. The residual stress caused by misfit in crystal lattice of film and substrate could influence conductivity. In our case, the dilatative strain in LaF_3_ could increase the conductivity along the strained region. Detailed RHEED analysis during the growing of the heterostructures showed a high quality of crystalline structure directly near the LaF_3_/SrF_2_ interfaces. In analysing the symmetry and lattice parameters of adjacent phases, we can expect extended lattice in the LaF_3_ interface region resulting in increased conductivity of heterostructure as in defect perovskites.[21] Another reason for conductivity enhancement might be the formation of La_1-x_Sr_x_F_3-x_ solid solution on the interface. We cannot exclude the possibility of the formation of solid solution on the boundary. Such a formation may result in the poor quality of our fit for thin layers, which may require an additional impurity profile near the boundary. More detailed analysis of the unexpected strong increase of the longitudinal conductivity in the heterostructures with thin layers (less than 25 nm) has not been performed yet.

## 4. Conclusions

LaF_3_/SrF_2_ multilayers with a large ionic conductivity have been grown and studied. The longitudinal conductivity of LaF_3_/SrF_2_ multilayer heterostructures with thicknesses of individual layers in the range 5–100 nm – along with films of pure LaF_3_ and SrF_2_ and solid solutions of La_0.95_Sr_0.05_F_2.95_ and La_0.5_Sr_0.5_F_2.5_ – have been measured by impedance spectroscopy in the frequency range 10^−1^–10^6^ Hz and a temperature range of 300–570 K. The use of multilayer structures allowed us to increase the conductivity of the thin films by three orders of magnitude compared with LaF_3_ films at room temperature (∽3∙10^−6^ S cm^–1^). The conductivity of LaF_3_/SrF_2_ multilayers showed a strong nonlinear increase (~100 times) when the interfacial spacing was decreased to 25 nm. A further decrease of the interfacial spacing to 5 nm reduced the conductivity, probably because of the overlap of the interfacial layers and the influence of film roughness. The dependence of the conductivity of layered structures on the thickness has been analysed in the framework of a theoretical approach describing a redistribution of charge carriers on the interface due to different chemical potentials. For large interfacial spacing (down to 50 nm) the qualitative description of conductivity growth has been obtained. However, in the materials under study one can envision additional mechanisms of interface conductivity growth. These mechanisms have probably contributed along with interface strain due to lattice mismatch between LaF_3_ and SrF_2_ and/or formation of a very thin solid-solution layer on the interface having higher conductivity.

## Disclosure statement

No potential conflict of interest was reported by the authors.

## Funding

The work at SPbPU was performed under the government order of Ministry of Education and Science of the Russian Federation.
